# Decrease in household secondhand smoking among Korean adolescents associated with smoke-free policies: grade-period-cohort and interrupted time series analyses

**DOI:** 10.4178/epih.e2024009

**Published:** 2023-12-13

**Authors:** Hana Kim, Heewon Kang, Sung-il Cho

**Affiliations:** 1 Department of Public Health Sciences, Graduate School of Public Health, Seoul National University, Seoul, Korea; 2 Institute of Health and Environment, Seoul National University, Seoul, Korea

**Keywords:** Age-period-cohort analysis, Interrupted time series analysis, Segmented regression, Secondhand smoking, Adolescents

## Abstract

**OBJECTIVES:**

Smoke-free areas have expanded and related campaigns have been implemented since 1995 in Korea. As a result, household secondhand smoke (SHS) exposure has decreased over the past 15 years. We assessed the cohort effect, the effect of a 2008 campaign on household SHS exposure, and the impact of a complete smoking ban in public places along with increased penalties, as implemented in December 2011.

**METHODS:**

Nationally representative cross-sectional 15-wave survey data of Korean adolescents were used. The 810,516 participants were classified into 6 grade groups, 15 period groups, and 20 middle school admission cohorts. An age-period-cohort analysis, conducted with the intrinsic estimator method, was used to assess the cohort effect of household SHS exposure, and interrupted-time series analyses were conducted to evaluate the effects of the smoke-free policy and the campaign.

**RESULTS:**

For cohorts who entered middle school from 2002 to 2008, the risk of household SHS exposure decreased among both boys and girls. Immediately after implementation of the smoke-free policy, the prevalence of household SHS exposure by period decreased significantly for boys (coefficient, -8.96; p<0.05) and non-significantly for girls (coefficient, -6.99; p=0.07). After the campaign, there was a significant decrease in household SHS exposure by cohort among boys, both immediately and post-intervention (coefficient, -4.84; p=0.03; coefficient, -1.22; p=0.02, respectively).

**CONCLUSIONS:**

A school-admission-cohort effect was found on household SHS exposure among adolescents, which was associated with the smoke-free policy and the campaign. Anti-smoking interventions should be implemented consistently and simultaneously.

## GRAPHICAL ABSTRACT

[Fig f3-epih-46-e2024009]

## Key Message

Household secondhand smoke (SHS) exposure among Korean adolescents has decreased over the past 15 years. There was a school admission cohort effect on household SHS exposure. A complete smoking ban in public space with increased penalties in December 2011 and a campaign in 2008 to avoid SHS exposure were associated with reduced household SHS exposure among adolescents.

## INTRODUCTION

As recognized by the Charter of the World Health Organization (WHO) and the United Nations Convention on the Rights of the Child, protecting adolescents from secondhand smoke (SHS) is necessary to uphold their right to life and to maintain the highest possible level of health [1]. SHS exposure could increase morbidity and cause premature death in children [2]. From 2010 to 2018, the prevalence of household SHS exposure among adolescents aged 12-16 years across 142 countries was 33.1% for more than 1 day a week, 20.1% for more than 3 days, and 12.3% for each day [3].

The WHO Framework Convention on Tobacco Control (FCTC)-related smoke-free policies and campaigns need to be monitored. According to Article 8 of the WHO FCTC, smoking in all indoor workplaces, on public transport, and in public places should be prohibited to prevent SHS exposure [4,5]. In 2020, the average implementation rate of Article 8 was 85%, the highest among all FCTC provisions [6]. Article 12 of the WHO FCTC proposed guidelines to enhance the effectiveness of communication, education, and training aimed at raising public awareness about tobacco control [7]. The average implementation rate of Article 12 in 2020 was 76%, behind those of Articles 8 (85%) and 11 (81%) [6].

Media campaigns have significantly enhanced public awareness and reduced household SHS exposure [8]. For instance, hospitalization rates due to asthma among children < 5 years old declined after the 2014 mass media campaign “Take it Right Outside” in Scotland [9]. In Taiwan, the prevalence of household SHS exposure among adults decreased from 36.8% to 34.3% after a media campaign in 2008 [8].

In Korea, household SHS exposure among adolescents significantly decreased from 40.3% to 25.4% from 2006 to 2020 [10]. Before and during this period, smoke-free area policies and media campaigns were implemented [11-13]. Since the National Health Promotion Act of 1995, the Korean government has gradually expanded indoor smoke-free areas in public facilities (Supplementary Material 1). Smoking was partially banned in public baths (1999), game rooms (2003), and indoor workplaces (2006) [11]. Since the National Health Promotion Act was revised in 2011, a complete smoking ban with increased penalties has applied to schools, kindergartens, and medical facilities (2011), all restaurants (2015), indoor sports facilities (2017), and food vending machines in indoor places (2019) [11,12]. Despite this expansion of policies, Korea was considered to have insufficiently implemented smoke-free policies by the WHO FCTC [14] because indoor smoking rooms were allowed in other smoke-free areas, except in medical and educational institutions, and the technical requirements of the WHO were unmet [15].

Korea was considered by the WHO FCTC to have met all conditions for anti-tobacco mass media campaigns [15]. A public advertising campaign was implemented in 2000 [11]. The “Say No, Save Lives” campaign, which highlighted the importance of speaking out about the right not to be exposed to SHS, was launched in 2008 [13]. The expansion of smoke-free areas was promoted to create a new social norm, from 2011 to 2012 and then again in 2017 [11].

Changes in social norms have been induced by tobacco control policies and campaigns [16,17]. Cohort effects can be used to detect changes in social norms, and trends (such as smoking prevalence) should be studied in the context of cohorts that share generational values [16]. Adolescents are more receptive to social pressures than adults [18] but are exposed to SHS in areas such as their houses, cars, schools, and public places [19]. Public places, including schools, can be directly regulated by designating smoke-free areas, whereas houses are difficult to regulate because they are private spaces [20]. However, designating smoke-free areas in public places could promote smoke-free homes by shifting social norms related to the risk of SHS exposure [21]. Following the implementation of smoke-free legislation, there was an increase in households that imposed smoking bans, in agreement with the social diffusion hypothesis [21,22].

To our knowledge, no studies have assessed school admission cohort effects on household SHS exposure among adolescents. We analyzed school admission cohorts instead of birth cohorts because strengthening tobacco control measures and promoting negative attitudes related to smoking could influence adolescents entering middle school, who constitute the largest group of persons initiating smoking [23]. Moreover, previous studies used short-term data and small samples [8,21], whereas our findings are based on a large sample and cover a period of 15 years.

Smoke-free policies and mass media campaigns can indirectly affect SHS exposure by influencing smoking cessation and social norms [24]. We aimed to assess the school admission cohort effect on household SHS exposure among Korean adolescents and determine whether smoke-free policies and campaigns were associated with a reduction in household SHS exposure.

## MATERIALS AND METHODS

### Data source and participants

The Korea Youth Risk Behavior Survey (KYRBS), which is self-reported and anonymous, is conducted annually on middle-school and high-school students [10]. The average participation rate over 15 years was 95.8% (range: 90.9-97.7). Fifteen cross-sectional waves of survey data were used in this study, and 1,032,106 Korean adolescents participated in the surveys in the period 2006-2020. People who have never smoked were selected as the subjects [25], and 810,516 were eligible for inclusion in this study. We presented age distribution by grade (Supplementary Material 2).

### Measures

The outcome variable was defined using the question “How many days have you been at home when someone else (such as a family member or guest) smoked near you during the last 7 days?” Those who answered that they were exposed from 1 day to every day were classified as “exposed”; otherwise, they were classified as “unexposed.” Indicators of household SHS exposure were introduced in 2006, and there were subtle changes in the question in 2019 and 2020 [10]. In 2019, “when someone else smoked near you” was changed to “when you smelled cigarette smoke from someone else near you,” and “smelled” was changed again to “inhaled” in 2020. As such, caution is needed when comparing the prevalence of household SHS exposure.

### Statistical analysis

#### Age-period-cohort intrinsic estimator analysis

An age-period-cohort (APC) analysis enables investigation of the effects of health level and health inequality on long-term trends [26]. We conducted an APC analysis to evaluate the effect of the grade, period, and school admission cohort on household SHS exposure among Korean adolescents over 15 years [23]. Supplementary Material 3 shows household SHS exposure by period and school admission cohort stratified by grade. We distinguished 6 grade groups (grades 1-6: seventh to 12th), 15 survey period groups (2006-2020), and 20 middle-school admission cohorts (2001-2020), which were separated by 1-year intervals. The middle-school admission cohort was calculated as “survey period–grade+1.” The identification problem is caused by the perfect linear relationship of age (a), period (p), and cohort variables (c) (age= period−cohort) in APC analysis and can be corrected using the APC intrinsic estimator method [26]. We employed a log-linear model with a Poisson distribution for household SHS exposure. The reference school admission cohort was 2001, the reference survey year was 2006, and the reference grade was 1 (seventh). The equation used for APC analysis was as follows (1):


(1) ln[λ(a,p)]=f(a)+g(p)+h(c)

We evaluated the suitability of the grade-period-cohort model using residual deviation (Supplementary Material 4). Residual deviation is a statistical measure that indicates the degree of similarity between the predicted values of a model and the actual values of the data [27]. A lower residual deviation value indicates a higher level of precision in the model predictions [27].

#### Interrupted time series analysis-segmented regression

Interrupted time series (ITS) analysis is used to evaluate the effects of interventions, such as policies and programs, at the population level [28]. We used ITS analysis because randomized controlled trials, the gold standard for evaluating the effect of an intervention, are often infeasible for population-level health policies [28]. Because ITS analysis is typically carried out in a real-world setting, the data may be more robust and applicable on a wider scale [28].

We employed ITS analyses to assess the effects of two smoke-free policies. An ITS analysis of household SHS exposure by school admission cohort was conducted to assess the change caused by the 2008 “Say No, Save Lives” campaign. In addition, an ITS analysis of household SHS exposure by survey period was performed to determine the effects of the smoke-free regulations implemented in December 2011 (approximately 2012).

We analyzed the prevalence of household SHS exposure by survey period and school admission cohort. The grade-standardized prevalence of household SHS exposure by period was calculated based on the distribution of students participating in the 2006 KYRBS. We calculated the prevalence of household SHS exposure by school admission cohort using the number of students exposed to SHS as the numerator and the number of students as the denominator by school admission cohort. The standard population for the grade-standardized prevalence of household SHS exposure by cohort was obtained using the oldest school admission cohort for each grade. For example, for grade 6 (12th), the school admission cohort in 2001 was the oldest, and the school admission cohort in 2006 was the oldest for grade 1 (seventh).

Segmented regression analysis is a type of ITS analysis that enables calculation of the immediate change in slope for a given time series [29]. Segmented regression models fit a regression line to each segment of the independent variable and operate under the assumption of a linear relationship between the independent and dependent variables within each segment [30]. The R2 and adjusted R2 statistics were calculated to demonstrate the goodness-of-fit of the models [30]. The segmented regression model was as follows (2) [31]:


(2) $$Y=b_{0}+b_{1}\times [time]+b_{2}\times [policy]+b_{3}\times [time\;\sin ce\;policy]+\varepsilon$$

*Y: grade-standardized prevalence of household SHS exposure among adolescents; time: period (1-15) and cohort (1-20); policy: 0,1; time since policy: time in years after policy implementation; ε: error term.*

We also plotted the counterfactual scenario. The counterfactual scenario is a hypothetical scenario in which interventions, such as policies and campaigns, did not happen such that the trend of interest is unaltered [28]. We assumed a counterfactual scenario in which the smoke-free policy and the campaign had not been implemented. Counterfactual scenarios were generated for 2008 (launch of the campaign) and 2012 (implementation of the smokefree policy), and we assumed that the change in policy and time since policy both had values of 0 (3) [31]:


(3) $$Y=b_{0}+b_{1}\times [time]+b_{2}\times 0+b_{3}\times 0+\varepsilon$$

*Y: grade-standardized prevalence of household SHS exposure among adolescents; time: period (7) and cohort (8); policy: 0; time since policy: 0; ε, error term.*

We used 3 methods to assess the autocorrelations of residuals, which could induce overestimation of the impact of the intervention [29]. We generated an autocorrelation function plot (Supplementary Material 5), conducted the Breusch-Godfrey test, and the Durbin-Watson test [28,32]. Statistical analysis was performed using SAS version 9.4 (SAS Institute Inc., Cary, NC, USA) and R version 4.2.2 (R Foundation for Statistical Computing, Vienna, Austria).

### Ethics statement

This analysis of secondary data was exempt from the requirement for approval by the Institutional Review Board of Seoul National University (IRB No. E2109/003-005).

## RESULTS

**Figure 1.**
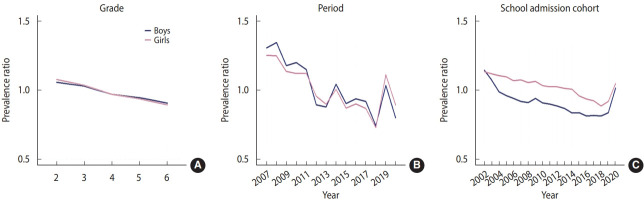
Estimated grade (A)-period (B)-school admission cohort (C) effects for secondhand smoking exposure in the home.

The fitting results of the 5 APC models are shown in Supplementary Material 4. The full APC model showed the best fit for both genders. The grade, period, and cohort effects of household SHS exposure among adolescents are shown in Figure 1, and estimates are provided in Supplementary Material 6. The predicted prevalence ratio (PR) for the grade effect decreased as grade increased. The period effect was significant for both genders and showed similar patterns. With the exception of 2013 and 2014, the PR decreased from 2008 (boys: 1.341; girls: 1.245) to 2018 (boys: 0.743; girls: 0.730). The estimated PR increased from 2018 to 2019 and decreased after 2020. The PR was > 1 from 2008 to 2011 but decreased to < 1 after 2012.

The school admission cohort effect showed decreasing PRs for both genders. Between 2002 (boys: 1.143; girls: 1.131) and 2008 (boys: 0.908; girls: 1.054), the PR of household SHS exposure decreased in the school admission cohorts. The PR continued to decrease for girls from 2009 (1.062) to 2018 (0.884) but increased from 2018 (0.884) to 2020 (1.046). Boys who entered middle school between 2009 (0.940) and 2014 (0.834) had a lower PR of household SHS exposure, but those who entered middle school after 2018 had a significantly increased PR.

The results of the segmented regression analysis are presented in Table 1. No significant autocorrelation effect was observed, except for the prevalence of SHS household exposure by school admission cohort among girls. For boys, a slightly increasing trend by period was observed, which was not significant before 2012. There was an immediate effect only after the smoke-free policy was implemented in 2012. The policy did not exert a significant persistent effect on the grade-standardized prevalence of household SHS exposure by period after 2012, but the direction of the slope differed from the counterfactual scenario. For girls, the slope change was not significant before the policy (2006-2011), at the time of its implementation (2012), or after the policy (2013-2020). Therefore, the smoke-free policy implemented in December 2011 did not significantly decrease household SHS exposure among girls.

The effect of the “Say No, Save Lives” campaign in 2008 was evaluated by calculating the grade-standardized prevalence of household SHS exposure by school admission cohort. For boys, the slope change of the time series was not significant from 2001 to 2007. However, there was a significant immediate reduction in 2008 and a persistent reduction from 2009 to 2020. For girls, a significant decreasing trend was identified after the implementation of the campaign, but autocorrelation of the residuals was confirmed.

**Figure 2.**
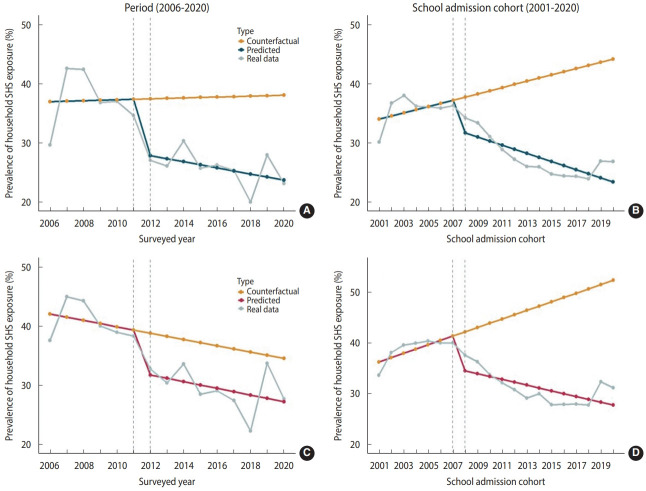
Counterfactual scenarios by period and school admission cohort. (A) Boys: complete smoking ban in public space with increased penalties in December 2011. (B) Boys: ‘Say No, Save Lives’ campaign in 2008. (C) Girls: complete smoking ban in public space with increased penalties in December 2011. (D) Girls: ‘Say No, Save Lives’ campaign in 2008.

Counterfactual scenarios in which the policy and the campaign were not introduced in the same setting are shown in Figure 2. For boys, the grade-standardized prevalence of household SHS exposure over 15 years would have increased slightly if the smoke-free policy had not been introduced in 2012. For girls, the grade-standardized prevalence of household SHS exposure over 15 years would have decreased in the counterfactual scenario. We predicted an increasing trend in the grade-standardized prevalence of household SHS exposure by school admission cohort in the counterfactual scenario.

## DISCUSSION

We found a school admission cohort effect of household SHS exposure among Korean adolescents and an effect of the 2008 “Say No, Save Lives” campaign and the complete smoking ban in public places implemented in December 2011. The trend in PR by school admission cohort for boys decreased to a greater extent than for girls. The smoke-free policy immediately and significantly decreased household SHS exposure in boys, but not in girls. Trends in household SHS exposure by school admission cohort among boys showed variations depending on whether the campaign was implemented.

Cohorts who recently entered middle school had a lower risk of household SHS exposure. This may be due to a decrease in smoking prevalence as a result of the strengthening of tobacco control policies over time in Korea. These government-led tobacco control policies have significantly decreased smoking prevalence among men [12] and encouraged the adoption of smoke-free home rules [21]. Household SHS exposure among children decreased after a smoking ban was introduced in public places [33]. Therefore, recent school admission cohorts may experience lower household SHS exposure because of an increased number of non-smoking families and the establishment of a social norm against SHS.

The school admission cohort effect differed by gender, with a risk reduction seen for boys, but not girls. In addition, the effects of the smoke-free policy and the campaign were significant for boys, and the effect of the campaign was significant for girls, although autocorrelation of residuals was seen for the latter group. We explain this gender difference as follows. First, the lower SHS exposure among boys compared to girls might be associated with less frequent conversations with smoking fathers [34]. Korean girls might be more exposed to household SHS due to their close relationships and frequent conversations with their fathers, who are the primary source of SHS [34,35]. Second, the gender disparity in the time spent indoors at home could be attributable to differences in SHS exposure by gender. According to Rye et al. [36], Korean girls attending middle and high schools spent more time indoors at home than boys on weekdays. Girls, who tend to stay at home more, might have been more vulnerable to SHS because of smoking by a family member in the home. These may explain the greater decrease in household SHS exposure among boys than girls.

We found that the PR by period effect first decreased and then increased in 2019. This might be a consequence of the broader definition of SHS. The question inquiring about proximity to someone smoking was changed to one asking about the experience of smelling or inhaling tobacco smoke, which could have led to overestimation [37]. Our APC analysis showed that the risk of household SHS exposure decreased as grade increased among both boys and girls. This can be explained by a characteristic of the educational environment in Korea: senior students are less likely to be exposed to SHS at home because they spend more time in class and have less contact with their parents [34]. This is because they are preparing for college entrance examinations [34].

The short-term effects of complete smoking bans in public places may not be sufficient to identify changes in household SHS exposure, given the ongoing designation of partial smoke-free areas. Political effort and time may be needed for a smoking ban in public places to lead to smoke-free homes. Nonetheless, the slope of the trend in household SHS exposure was negative compared to the counterfactual scenario.

Our findings suggest that the reduction in household SHS exposure among school admission cohorts could have been affected by social norms. The campaign induced a significant slope change and a decrease in SHS exposure for school admission cohorts among boys. A significant decreasing trend occurred among girls’ school admission cohorts after the intervention, but there was autocorrelation in the residuals. The “Say No, Save Lives” campaign emphasized the importance of talking about the rights of people who do not smoke [13]. Claiming these rights can serve as a social control, possibly prompting smokers to comply with smoke-free rules [38].

As a result of the influence of a 2008 campaign, cohorts entering middle school after 2008 were less exposed to household SHS, due to decreasing smoking prevalence among adults [39]. Changes in social norms affect behavior at both the individual and population levels [40]. Consistent monitoring of smoke-free areas and public awareness thereof is important to increase compliance with smoke-free policies [41]. After the campaign, the continued expansion of smoke-free areas and campaigns lowered exposure among students entering middle school after 2008.

We investigated the effects of 2 interventions. The policy targeting the total population and the campaign targeting vulnerable groups such as children and women, exerted different effects. However, these interventions may not have been directly associated with the decline in household SHS exposure because they targeted public places rather than homes. There was no decrease in parental smoking at home in Macao after a smoking ban in public places [42]. According to the displacement hypothesis, the likelihood of parental smoking at home and household SHS exposure among children would be increased if smoking parents could no longer smoke in public places [22,42,43]. These points need to be borne in mind when interpreting our results.

This study was novel in that we identified associations between a smoke-free policy and a campaign targeting public places with a reduction of SHS exposure in personal spaces (such as homes) among adolescents. In addition, we discovered that the smoke-free campaign had a generational impact. We separately assessed the effects of the campaign and the smoke-free policy. We suggest implementing these interventions concurrently or sequentially because this approach is associated with a greater reduction in household SHS exposure than a single intervention [8]. This has implications for policymakers regarding the continuing implementation of smoke-free policies and campaigns.

This study had several limitations. First, only the observed changes can be interpreted because of the design and technical aspects of the APC model [16]. Second, because out-of-school adolescents did not participate in the KYRBS [44], their household SHS exposure was not evaluated. Third, data on smoking history and household SHS exposure among adolescents may have been more accurate had serum cotinine been measured [45,46], because the KYRBS data are self-reported. Fourth, we report associations rather than causal relationships, as with any observational study [9]. Fifth, other tobacco control policies such as tax increases and health warning labeling might have influenced the declining trends [47,48], although neither intervention involved substantial changes in other policies. Lastly, the impact of the intervention might have been overestimated as a result of the autocorrelation of the residuals [29].

In conclusion, there was a school admission cohort effect on household SHS exposure among Korean adolescents. A complete smoking ban in public places with increased penalties and a campaign to avoid SHS exposure were associated with reduced household SHS exposure among adolescents. To prevent household SHS exposure among adolescents, appropriate social norms need to be established, and effective policies and campaigns implemented together.

## Figures and Tables

**Figure f3-epih-46-e2024009:**
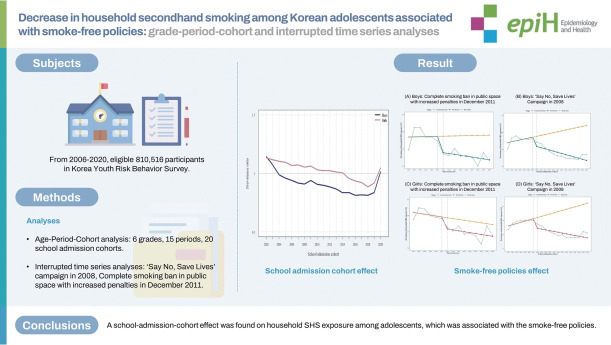


**Table 1.** Interrupted time series analyses of secondhand smoking (SHS) at home among adolescents in Korea

Time series	Prevalence of household SHS exposure by period (2006-2020)				Prevalence of household SHS exposure by school admission cohort (2001-2020)			
Intervention	Complete smoking ban in public space with increased penalties in December 2011				“Say No, Save Lives” campaign in 2008			
Effect	Boys	p-value	Girls	p-value	Boys	p-value	Girls	p-value
Pre-intervention	0.08 (0.94)	0.94	-0.53 (0.82)	0.53	0.53 (0.43)	0.23	0.84 (0.43)	0.07
Immediate effect	-8.96 (4.04)	<0.05	-6.99 (3.54)	0.07	-4.84 (2.04)	0.03	-6.27 (2.05)	<0.01
Post-intervention	-0.60 (1.07)	0.59	-0.03 (0.94)	0.97	-1.22 (0.46)	0.02	-1.40 (0.46)	<0.01
Constant	36.88 (3.67)	<0.001	42.48 (3.21)	<0.001	33.51 (1.92)	<0.001	35.36 (1.92)	<0.001
Model								
R^2^/adjusted R^2^	0.74/0.67		0.78/0.72		0.83/0.79		0.81/0.77	
p-value	<0.01		<0.001		<0.001		<0.001	

Values are presented as coefficient (standard error).

## Supplementary materials

Supplementary Material 1. Smoke-free air policies and campaigns in Korea (1995–2020)

Supplementary Material 2. Age distribution by grade.

Supplementary Material 3. Household SHS exposure by period and school admission cohort stratified by grade among Korean boys and girls.

Supplementary Material 4. Goodness of fit for each model.

Supplementary Material 5. ACF plot of residuals.

Supplementary Material 6. Prevalence ratio for grade, period, school admission cohort by gender
