# Supplementary material for: Decrease in household secondhand smoking among Korean adolescents associated with smoke-free policies: grade-period-cohort and interrupted time series analyses
Source: Epidemiol Health. 2023 Dec 13;46:e2024009. doi: 10.4178/epih.e2024009 (PMC11040220; doi:10.4178/epih.e2024009)
Supplement: Supplementary Material 1. — Smoke-free air policies and campaigns in Korea (1995–2020) [file epih-46-e2024009-Supplementary-1.docx]

**Supplement 1. Smoke-free air policies and campaigns in Korea (1995–2020)**

| 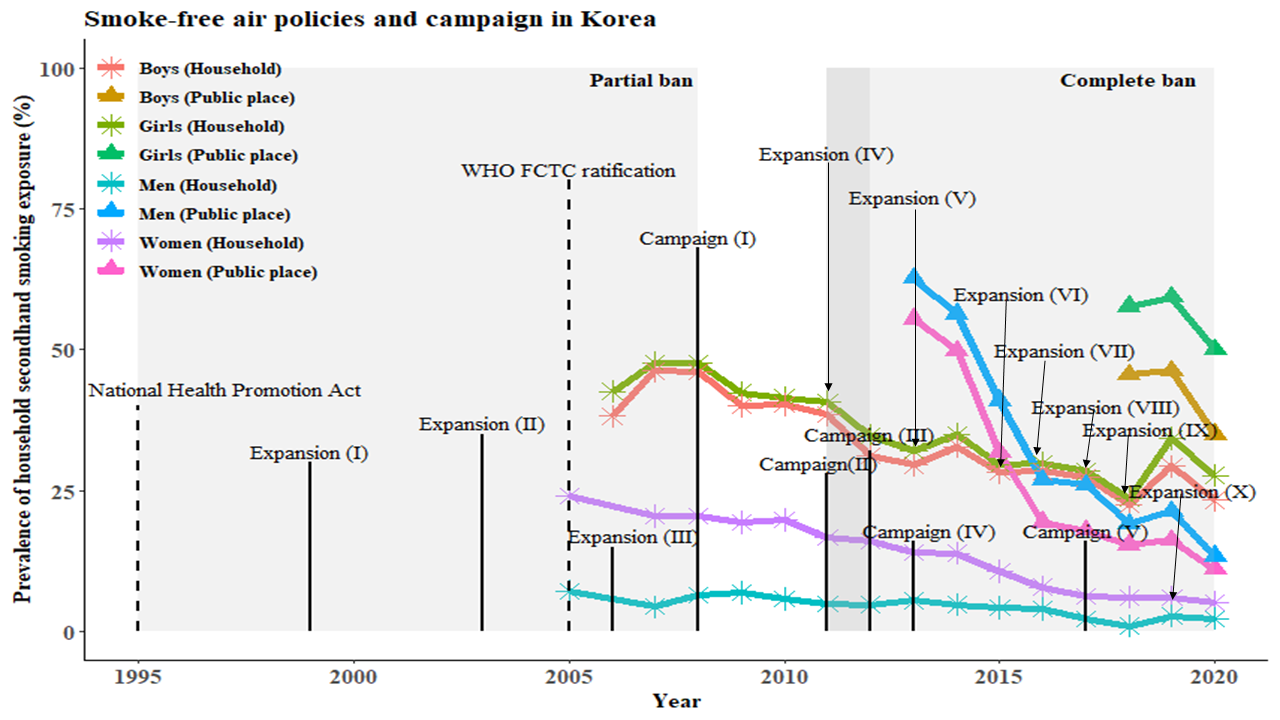  †The survey item asking about household SHS exposure among Korean adolescents changed in 2019 and 2020. |
| --- |
| **Smoke-free policies in the National Health Promotion Act**  ***Partial ban***  1995. National Health Promotion Act: first designation of smoke-free areas  1999. Expansion (Ⅰ): public baths  2003. Expansion (Ⅱ): game rooms, restaurants that does not sell alcoholic beverage (≥150m^2^), comic book stores, government buildings (≥1,000m^2^), nursery schools  2006. Expansion (Ⅲ): factories, local government buildings, indoor workplaces  ***Complete ban***  2011. Expansion (Ⅳ): schools, kindergartens, daycare centers, and medical facilities with penalties  2013. Expansion (Ⅴ): restaurants (≥100m^2^)  2015. Expansion (Ⅵ): all restaurants  2016. Expansion (Ⅶ): multi-family housing  2017. Expansion (Ⅷ): indoor sports facilities  2018. Expansion (Ⅸ): within 10 meters of kindergartens, child care facilities  2019. Expansion (Ⅹ): food vending machines in indoor places  2020. Recommendation for designation of outdoor smoking areas  **Ministry of Health and Welfare national campaigns related to secondhand smoke (SHS) exposure prevention**  2008. Campaign (Ⅰ): ‘Say No, Save Lives’  2011. Campaign (Ⅱ): ‘No smoking signs? Still No Smoking’  2012. Campaign (Ⅲ): ‘Look! Healthy smoke-free areas in Korea’  2013. Campaign (Ⅳ): ‘Everywhere Smoke-free Zone’  2017. Campaign (Ⅴ): ‘Expanding smoke-free areas’ |

**References**

1. Ministry of Health and Welfare, Korea Health Promotion Institute. 2021 Guidance on Community Integrated Health Promotion Project [Smoking cessation]. 2020 (Korean, author's translation).
2. Kwak J, Jeong H, Chun S, Bahk JH, Park M, Byun Y, et al. Effectiveness of government anti-smoking policy on non-smoking youth in Korea: a 4-year trend analysis of national survey data. BMJ open. 2017;7(7):e013984.
3. Park J, Minh LN, Shin SH, Oh JK, Yun EH, Lee D, et al. Influence of new tobacco control policies and campaigns on Quitline call volume in Korea. Tobacco Induced Diseases. 2019;17:21.
4. Easy to find, Practical Law. Smoking cessation promotion and campaign. [cited 2023 August 1]. Available from: https://easylaw.go.kr/CSP/CnpClsMain.laf?popMenu=ov&csmSeq=908&ccfNo=4&cciNo=2&cnpClsNo=1.
5. Korea Disease Control and Prevention Agency. Korea National Health and Nutrition Examination Survey. [cited 2023 December 08]. Available from: https://knhanes.kdca.go.kr/knhanes/main.do.
6. Korea Disease Control and Prevention Agency, Ministry of Education, Ministry of Health and Welfare. Korea Youth Risk Behavior Survey. [cited 2023 December 08]. Available from: https://www.kdca.go.kr/yhs/.
