# Supplementary material for: Decrease in household secondhand smoking among Korean adolescents associated with smoke-free policies: grade-period-cohort and interrupted time series analyses
Source: Epidemiol Health. 2023 Dec 13;46:e2024009. doi: 10.4178/epih.e2024009 (PMC11040220; doi:10.4178/epih.e2024009)
Supplement: Supplementary Material 2. — Age distribution by grade. [file epih-46-e2024009-Supplementary-2.docx]

**Supplement 2. Age distribution by grade.**

| **Grade** | **N^*^** | **Mean (SD)** | **Median (Min, Max)** |
| --- | --- | --- | --- |
| Grade 1 (Middle 1^st^, 7^th^) | 156,505 | 12.56 (0.52) | 12.10 (12, 18) |
| Grade 2 (Middle 2^nd^, 8^th^) | 146,934 | 13.54 (0.52) | 13.07 (12, 18) |
| Grade 3 (Middle 3^rd^, 9^th^) | 139,770 | 14.53 (0.52) | 14.05 (12, 18) |
| Grade 4 (High 1^st^, 10^th^) | 126,011 | 15.52 (0.53) | 15.03 (12, 18) |
| Grade 5 (High 2^nd^, 11^th^) | 121,409 | 16.50 (0.53) | 16.00 (12, 18) |
| Grade 6 (High 3^rd^, 12^th^) | 116,200 | 17.48 (0.53) | 16.97 (12, 18) |
| Total | 806,829 | 14.91 (1.76) | 14.35 (12, 18) |
| SD: standard deviation.  ^*^The number of participants was 810,516 and the age variable had 3,687 missing values. When evaluating age distribution by grade, only 806,829 data points were used. | | | |
