# Supplementary material for: Decrease in household secondhand smoking among Korean adolescents associated with smoke-free policies: grade-period-cohort and interrupted time series analyses
Source: Epidemiol Health. 2023 Dec 13;46:e2024009. doi: 10.4178/epih.e2024009 (PMC11040220; doi:10.4178/epih.e2024009)
Supplement: Supplementary Material 3. — Household SHS exposure by period and school admission cohort stratified by grade among Korean boys and girls. [file epih-46-e2024009-Supplementary-3.docx]

**Supplement 3. Household SHS exposure by period and school admission cohort stratified by grade among Korean boys and girls.**

| **Boys** | **Girls** |
| --- | --- |
| 1. 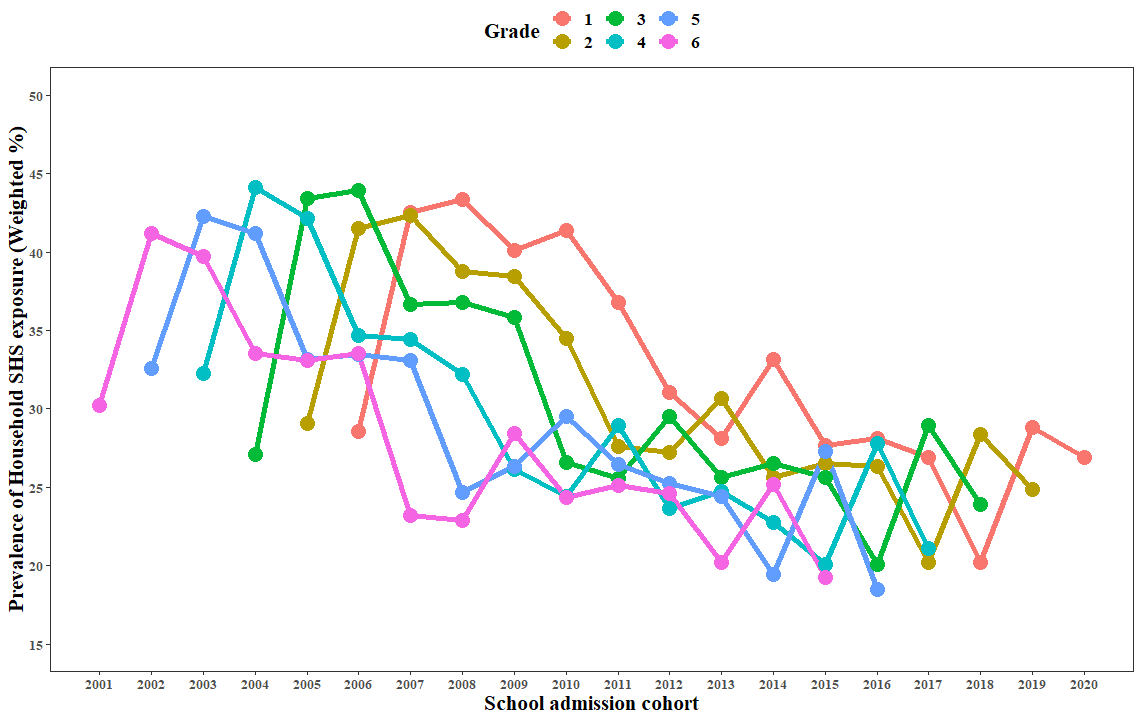School admission cohort ^*^ Grade | 1. 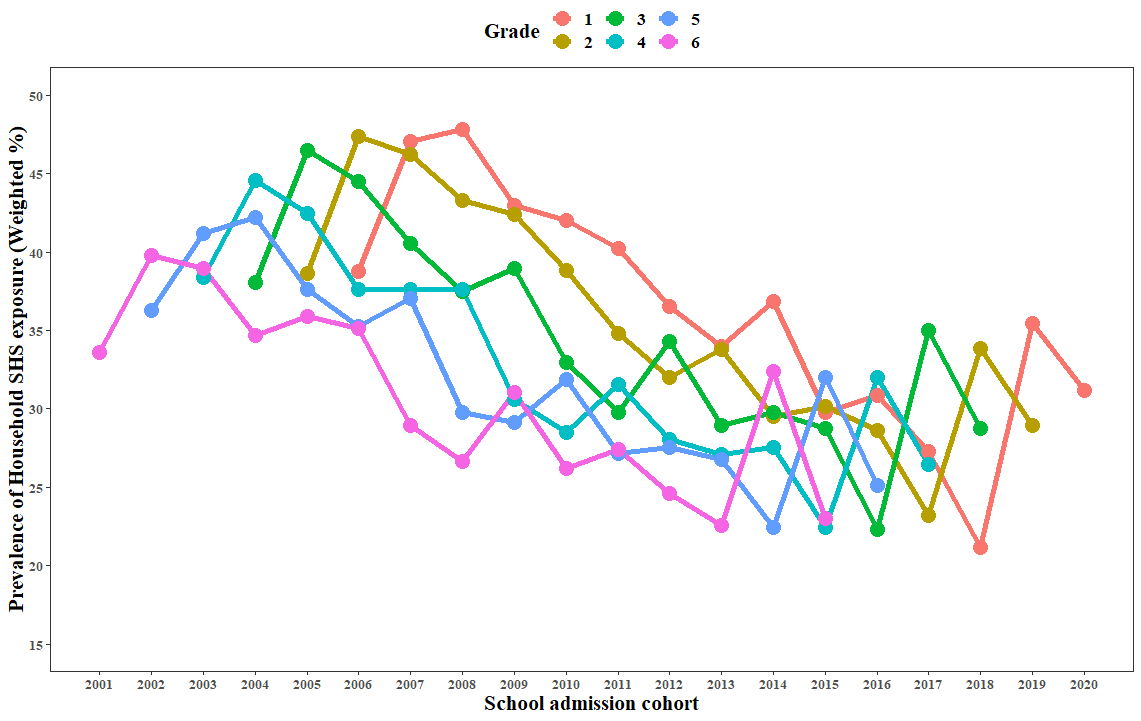School admission cohort ^*^ Grade |
| 1. 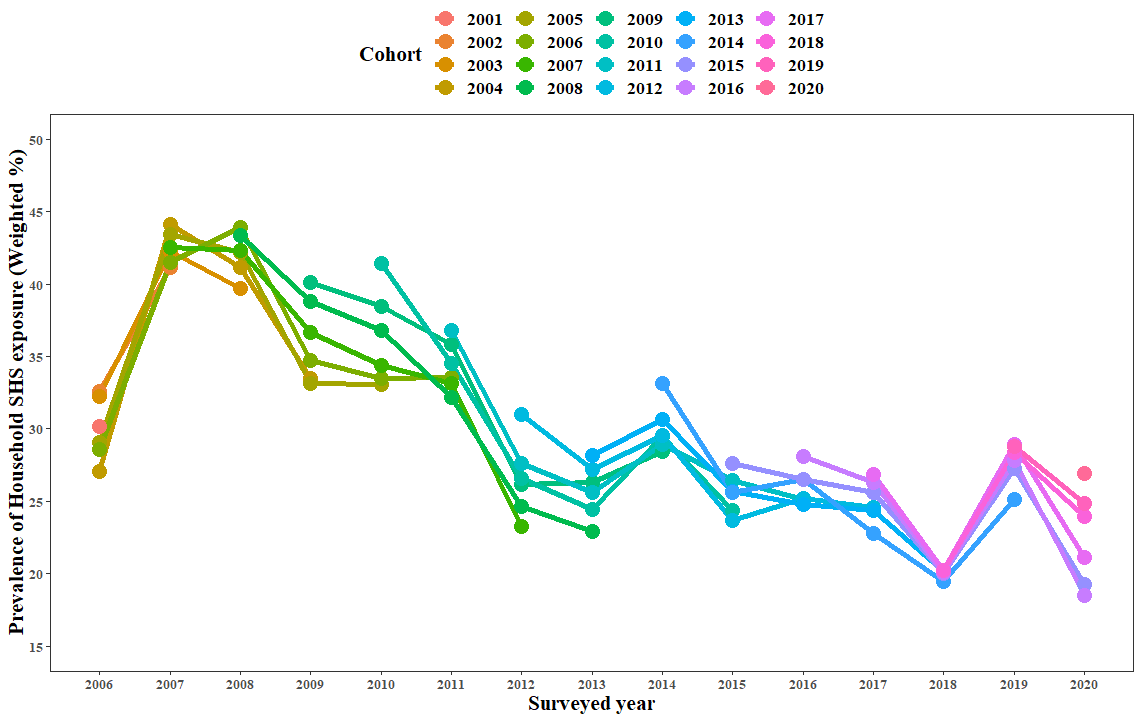Period ^*^ School admission cohort | 1. 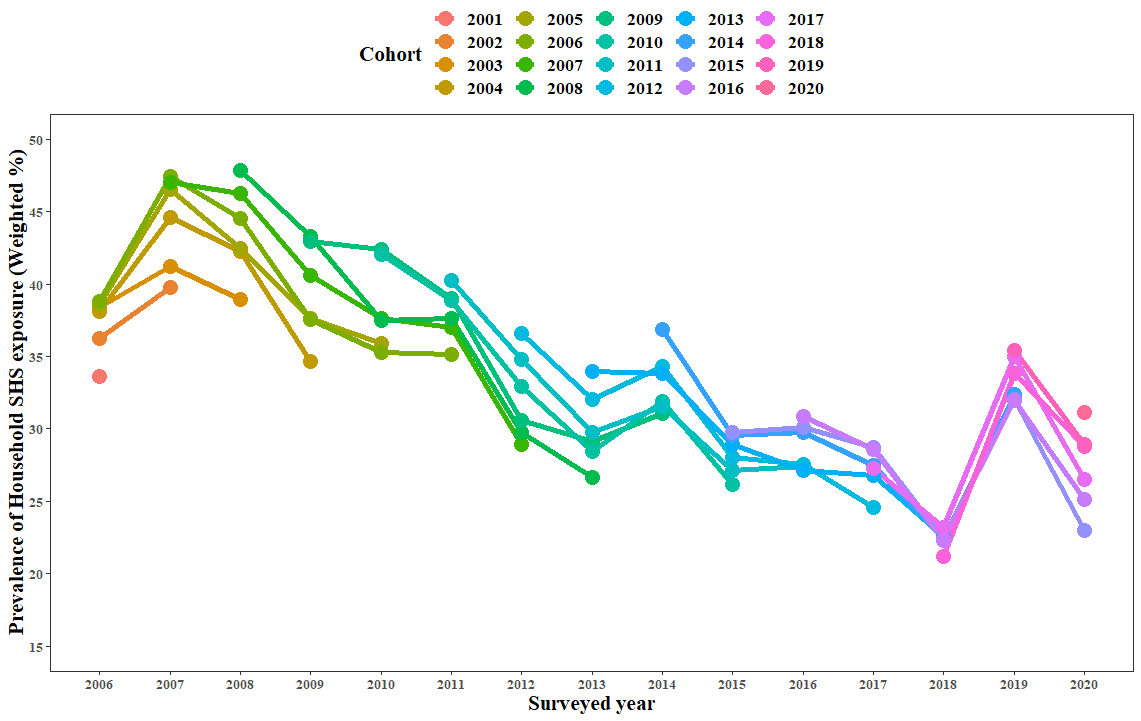Period ^*^ School admission cohort |
| 1. 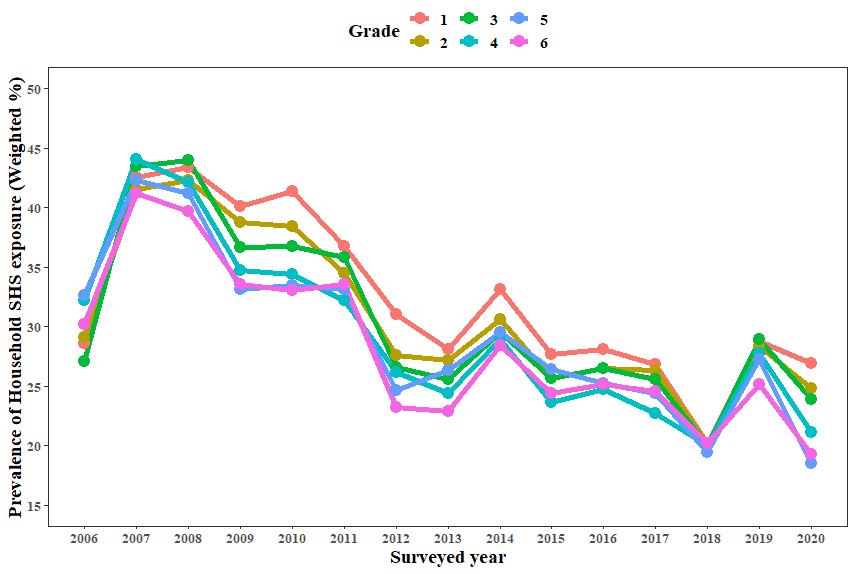 Period ^*^ Grade | 1. 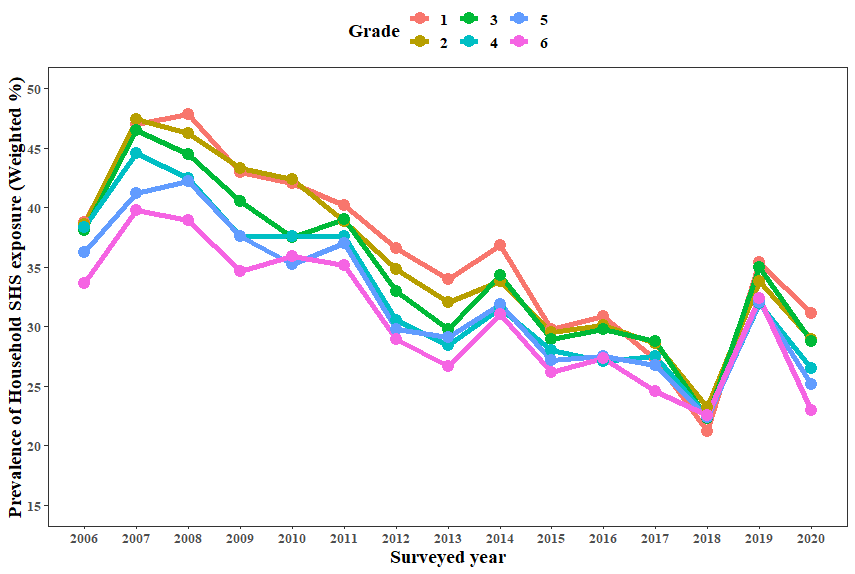Period ^*^ Grade |
