# Supplementary material for: Decrease in household secondhand smoking among Korean adolescents associated with smoke-free policies: grade-period-cohort and interrupted time series analyses
Source: Epidemiol Health. 2023 Dec 13;46:e2024009. doi: 10.4178/epih.e2024009 (PMC11040220; doi:10.4178/epih.e2024009)
Supplement: Supplementary Material 4. — Goodness of fit for each model. [file epih-46-e2024009-Supplementary-4.docx]

**Supplement 4. Goodness of fit for each model.**

| **Model** | **Boys** | | | | **Girls** | | | |
| --- | --- | --- | --- | --- | --- | --- | --- | --- |
|  | **AIC** | **Residual deviance (df)** | **Deviance difference (df)** | **p-value** | **AIC** | **Residual deviance (df)** | **Deviance difference (df)** | **p-value** |
| Grade | 287205.43 | 286035.31 (85) | - | - | 247123.64 | 245935.43 (85) | - | - |
| Grade-drift | 113987.58 | 112815.46 (84) | 173219.85 (1) | <.001 | 76815.08 | 75624.87 (84) | 170310.56 (1) | <.001 |
| Grade-School admission cohort | 108932.07 | 107753.95 (81) | 5061.51 (3) | <.001 | 70707.37 | 69511.16 (81) | 6113.71 (3) | <.001 |
| Grade-Period-School admission cohort | 65775.98 | 64591.86 (78) | 43162.09 (3) | <.001 | 53324.48 | 52122.27 (78) | 17388.89 (3) | <.001 |
| Grade-Period | 68421.80 | 67243.68 (81) | 2651.83 (3) | <.001 | 53648.71 | 52452.51 (81) | 330.24 (3) | <.001 |
| Grade-drift | 113987.58 | 112815.46 (84) | 45571.78 (3) | <.001 | 76815.08 | 75624.87 (84) | 23172.37 (3) | <.001 |
