# Supplementary material for: Decrease in household secondhand smoking among Korean adolescents associated with smoke-free policies: grade-period-cohort and interrupted time series analyses
Source: Epidemiol Health. 2023 Dec 13;46:e2024009. doi: 10.4178/epih.e2024009 (PMC11040220; doi:10.4178/epih.e2024009)
Supplement: Supplementary Material 5. — ACF plot of residuals. [file epih-46-e2024009-Supplementary-5.docx]

**Supplement 5. ACF plot of residuals.**

| **Period** | **School admission cohort** |
| --- | --- |
| 1. 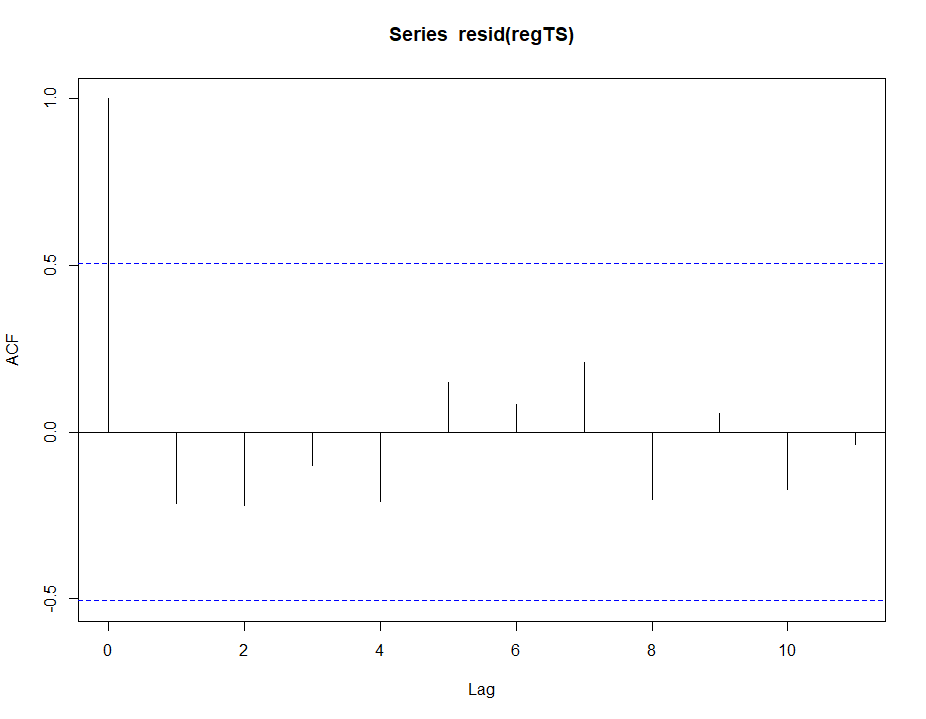Boys: Complete smoking ban in public space with increased penalties in December 2011. | 1. 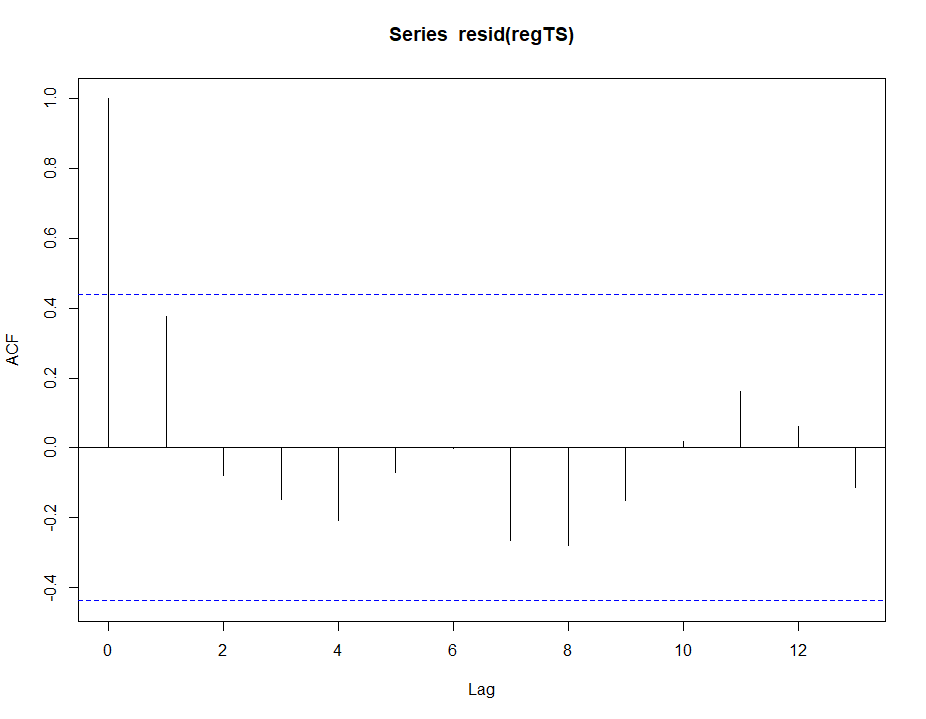Boys: ‘Say No, Save Lives’ campaign in 2008. |
| 1. Girls: Complete smoking ban in public space with increased penalties in December 2011.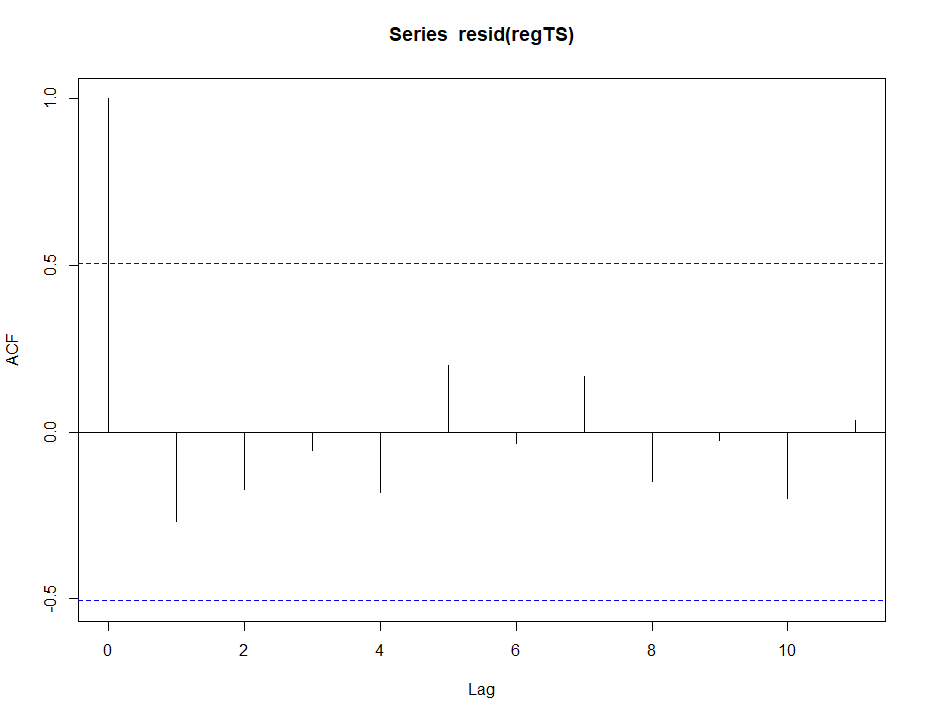 | 1. 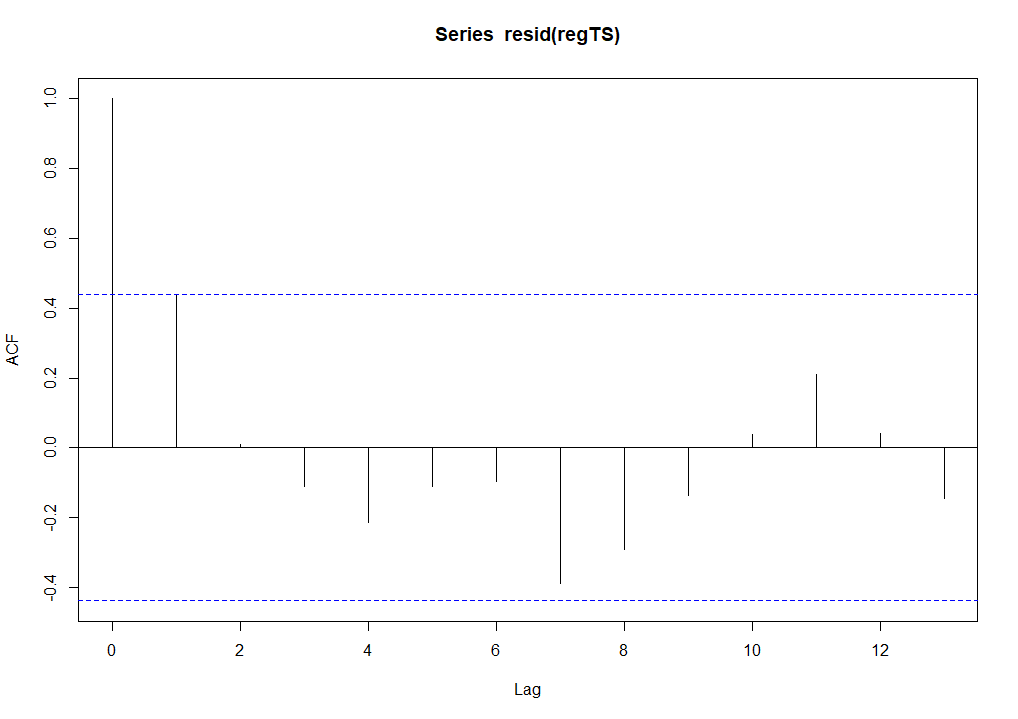Girls: ‘Say No, Save Lives’ campaign in 2008. |
