# Supplementary material for: Decrease in household secondhand smoking among Korean adolescents associated with smoke-free policies: grade-period-cohort and interrupted time series analyses
Source: Epidemiol Health. 2023 Dec 13;46:e2024009. doi: 10.4178/epih.e2024009 (PMC11040220; doi:10.4178/epih.e2024009)
Supplement: Supplementary Material 6. — Prevalence ratio for grade, period, school admission cohort by gender [file epih-46-e2024009-Supplementary-6.docx]

**Supplement 6. Prevalence ratio for grade, period, school admission cohort by gender**

| **Boys** | | **Girls** | |
| --- | --- | --- | --- |
| **Factor** | **Prevalence ratio** | **Factor** | **Prevalence ratio** |
| Grade 1 | 1 | Grade 1 | 1 |
| Grade 2 | 1.058 | Grade 2 | 1.077 |
| Grade 3 | 1.030 | Grade 3 | 1.036 |
| Grade 4 | 0.971 | Grade 4 | 0.971 |
| Grade 5 | 0.946 | Grade 5 | 0.938 |
| Grade 6 | 0.906 | Grade 6 | 0.894 |
| Period 1 | 1 | Period 1 | 1 |
| Period 2 | 1.302 | Period 2 | 1.250 |
| Period 3 | 1.341 | Period 3 | 1.245 |
| Period 4 | 1.175 | Period 4 | 1.133 |
| Period 5 | 1.197 | Period 5 | 1.119 |
| Period 6 | 1.148 | Period 6 | 1.120 |
| Period 7 | 0.895 | Period 7 | 0.956 |
| Period 8 | 0.879 | Period 8 | 0.898 |
| Period 9 | 1.041 | Period 9 | 1.005 |
| Period 10 | 0.904 | Period 10 | 0.871 |
| Period 11 | 0.937 | Period 11 | 0.901 |
| Period 12 | 0.917 | Period 12 | 0.868 |
| Period 13 | 0.743 | Period 13 | 0.730 |
| Period 14 | 1.034 | Period 14 | 1.109 |
| Period 15 | 0.800 | Period 15 | 0.892 |
| School admission cohort 1 | 1 | School admission cohort 1 | 1 |
| School admission cohort 2 | 1.143 | School admission cohort 2 | 1.131 |
| School admission cohort 3 | 1.072 | School admission cohort 3 | 1.117 |
| School admission cohort 4 | 0.987 | School admission cohort 4 | 1.103 |
| School admission cohort 5 | 0.959 | School admission cohort 5 | 1.095 |
| School admission cohort 6 | 0.939 | School admission cohort 6 | 1.066 |
| School admission cohort 7 | 0.917 | School admission cohort 7 | 1.072 |
| School admission cohort 8 | 0.908 | School admission cohort 8 | 1.054 |
| School admission cohort 9 | 0.940 | School admission cohort 9 | 1.062 |
| School admission cohort 10 | 0.906 | School admission cohort 10 | 1.032 |
| School admission cohort 11 | 0.898 | School admission cohort 11 | 1.023 |
| School admission cohort 12 | 0.885 | School admission cohort 12 | 1.024 |
| School admission cohort 13 | 0.867 | School admission cohort 13 | 1.012 |
| School admission cohort 14 | 0.834 | School admission cohort 14 | 1.006 |
| School admission cohort 15 | 0.838 | School admission cohort 15 | 0.957 |
| School admission cohort 16 | 0.813 | School admission cohort 16 | 0.936 |
| School admission cohort 17 | 0.817 | School admission cohort 17 | 0.922 |
| School admission cohort 18 | 0.812 | School admission cohort 18 | 0.884 |
| School admission cohort 19 | 0.837 | School admission cohort 19 | 0.918 |
| School admission cohort 20 | 1.015 | School admission cohort 20 | 1.046 |
